# Laser Welded versus Resistance Spot Welded Bone Implants: Analysis of the Thermal Increase and Strength

**DOI:** 10.1155/2014/357074

**Published:** 2014-07-03

**Authors:** Carlo Fornaini, Marco Meleti, Mauro Bonanini, Giuseppe Lagori, Paolo Vescovi, Elisabetta Merigo, Samir Nammour

**Affiliations:** ^1^Sezione di Odontostomatologia, Azienda Ospedaliero, Universitaria di Parma, Via A. Gramsci 14, 43100 Parma, Italy; ^2^Laser Unit, Dental Department, Faculty of Medicine, University of Liege, Belgium

## Abstract

*Introduction.* The first aim of this “ex vivo split mouth” study was to compare the thermal elevation during the welding process of titanium bars to titanium implants inserted in pig jaws by a thermal camera and two thermocouples. The second aim was to compare the strength of the joints by a traction test with a dynamometer. *Materials and Methods.* Six pigs' jaws were used and three implants were placed on each side of them for a total of 36 fixtures. Twelve bars were connected to the abutments (each bar on three implants) by using, on one side, laser welding and, on the other, resistance spot welding. Temperature variations were recorded by thermocouples and by thermal camera while the strength of the welded joint was analyzed by a traction test. *Results.* For increasing temperature, means were 36.83 and 37.06, standard deviations 1.234 and 1.187, and *P* value 0.5763 (not significant). For traction test, means were 195.5 and 159.4, standard deviations 2.00 and 2.254, and *P* value 0.0001 (very significant). *Conclusion.* Laser welding was demonstrated to be able to connect titanium implant abutments without the risk of thermal increase into the bone and with good results in terms of mechanical strength.

## 1. Introduction

The possibility of inserting titanium fixtures within the jawbones in order to replace missing teeth or as support for dental prostheses deeply changed the modern dentistry.

The modern researches in the field of dentistry include the performing of less aggressive surgical procedures, the reduction of the length of time of rehabilitation, and the possibility of obtaining a faster osseointegration. Moreover, there is an increasing scientific interest in the field of postoperative course in terms of faster tissues healing and better compliance of the patient [1, 2].

Broadly, implant surgical protocols can be classified into two distinguished techniques:

1. “transmucosal technique” also called “one-stage” surgery where the implant is not completely covered during the osseointegration phase and a small coronal portion of the fixture is left through the soft tissues within the oral cavity [3];
2. “submerged technique” also called “two-stage” surgery where the first phase consists in the complete positioning of the implant in the bone tissue and in its covering through soft tissues; during the period of osseointegration, which depends on the biomorphological characteristics of the implant surface and on the biology of the bone, the implant is completely covered; in the second surgical step (implant uncovering), there is the exposure of the coronal part of the fixture within the oral cavity [4].

In both techniques, there is today a great interest about the immediate loading possibility.

Donath et al. [5] reported that the load applied to the implant surface may interfere with the bone formation and it also may lead to fibrous encapsulation.

However, several clinical and experimental studies showed that long-term success of removable and fixed prostheses is possible also on immediately loaded implants [6, 7].

A clinical study based on a consistent number of implants showed the predictability and the high success percentage in immediately loaded cases [8] and the same authors demonstrated the absence of statistical differences, in terms of osseointegration and success percentage, between immediately and subsequently loaded implants, with a follow-up of 7 years [9]. The key factor connected to the success of the immediately loaded implants seems to be related to the absence of movement of 100 *μ*m or more just after their placement [10, 11]. Other studies indicated a micromovement of 150 *μ*m acceptable for a complete integration [12, 13].

Guruprasada et al. [14], by an “in vivo” study based on twenty patients, demonstrated that immediate implant loading protocol has a highly acceptable clinical success rate in partially edentulous lower jaw although implant survival rate is slightly inferior to conventional loading protocol while Stafford [15] concluded that there is no convincing evidence of a clinically important difference in prosthesis failure, implant failure, or bone loss associated with different loading times of implants.

Therefore, the possibility of immediately splinting the implant abutments just after their placement raised a very great interest [16].

At the moment only two techniques have been described as being able to weld metals intraorally, the Nd:YAG laser welding [17] and resistance spot welding [18].

The first aim of this “ex vivo split mouth” study was to compare the thermal elevation during the welding process of titanium bars to titanium implants inserted in pork jaws.

For this purpose, a thermal camera and two thermocouples were used, while, to analyze the strength of the two techniques, a dynamometer was employed.

The second aim was to compare the strength of the joint obtained by the two welding techniques by a traction test with a dynamometer.

## 2. Material and Methods

**Figure 1:**
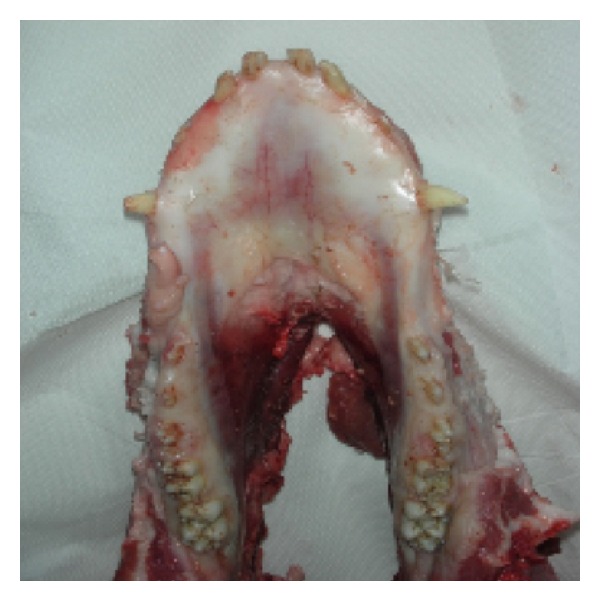
One of the specimens used for the tests.

Six pig jaws were used to carry out this study. Specimens were kept at room temperature and were used within 6 h of the animal sacrifice. As reported in the literature [19], specimens were stored during transit at 2–4°C and 100% of humidity to prevent tissue degradation (Figure 1).

All measurements were made at room temperature between 22°C and 24°C.

Mandibular tissues were macroscopically intact and the normal anatomical relationships were preserved.

Six implants, three on each side (Astra Tech, OsseoSpeed; *∅* 4 mm, length 11 mm), were placed in each anatomical specimen for a total of 36 fixtures.

The implant site was prepared through a micromotor (900 rpm).

All implants were placed with the most coronal loop completely inserted within the surrounding bone structure.

The placement of each implant was performed after the incision and the elevation of a mucoperiosteal flap followed by the exposure of the underlying bone. The implant site preparation was carried out through the execution of sequential steps with steel burs of increasing diameter (ranging from 2 mm to 3.7 mm) following the protocol indicated by the manufacturer (Astra Tech).

In order to evaluate the in-depth variations of temperature, two naked-bead chrome-aluminum (K-type) thermocouples (TP-01, Lutron, Taiwan) with a 0.5 mm diameter probe sensitive to temperature variations between −40°C and 250°C were placed; the thermocouples were connected to a two-channel thermometer (TM-946, Lutron, Taiwan) sensitive to temperature variations (for k-type thermocouples) between −100°C and 1,300°C, with an accuracy of 0.1°C.

A thermoconductor paste (Warme Leftpast WPN 10, Austerlitz Electronic, Germany) was spread on thermocouple probes to ensure good thermal contact; the thermal conductivity of the paste was 0.4 cal/s^−1^ m^−1^ K^−1^, in a way comparable with thermal conductivity of human tissues.

The thermocouples were used to measure temperature changes at bone level and on the fixture surface; The thermocouples were connected with two probes of 1.5 mm in diameter to measure simultaneously the temperature changes at bone level and on the fixture surface.

**Figure 2:**
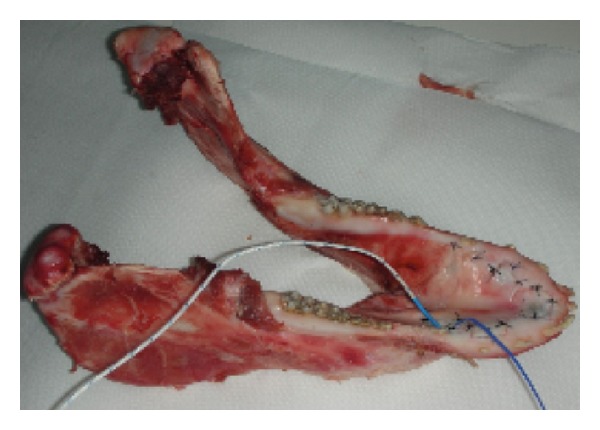
The two thermocouples inserted: one in the bone and the other in contact with the implant.

The thermal probes were placed within the bone tissue, through a path obtained with mini-implants surgical cutter (MDI IMTEC Corporation) of 1.1 mm in diameter (Figure 2).

The hole created was large enough both to contain the probe and to avoid fluctuations to thus induce possible alterations during the test.

The first probe was placed parallel to the long axis of the fixture, at a distance of 3 mm from its surface and at a depth of 4 mm from the margin of the bone. Such a probe was used to measure the peri-implant thermal changes (temperature at bone level: TB). The second probe was placed in contact with the fixture till a depth of 3 mm in the bone following an oblique path. Such a probe was used to evaluate the thermal variations on the implant (temperature at implant surface level: TS).

**Figure 3:**
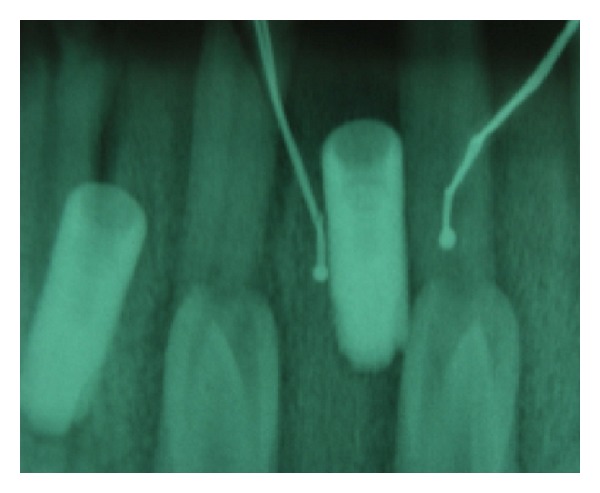
Rx check of the two thermocouples' position.

Radiographs were used to check the correct position of the probes on each site (Figure 3).

After the placement of the fixtures and the positioning of the probes, the flaps were sutured with interrupted sutures technique, using silk thread 4.0.

Surface temperature was checked during all procedures with a thermal camera (ThermoVision A 800, FLIR Systems, Stockholm, Sweden) connected to a PC and working with the Software ThermaCam Researcher; this thermal camera device has a 320 × 240 pixel detector with a resolution of 0.08°C. The infrared camera is a reliable and reproducible technique that allows noncontact high-resolution monitoring surface temperature in different medical applications, such as monitoring temperature during surgical procedure, or uses the thermal evaluation as diagnostic tool [20, 21].

**Figure 4:**
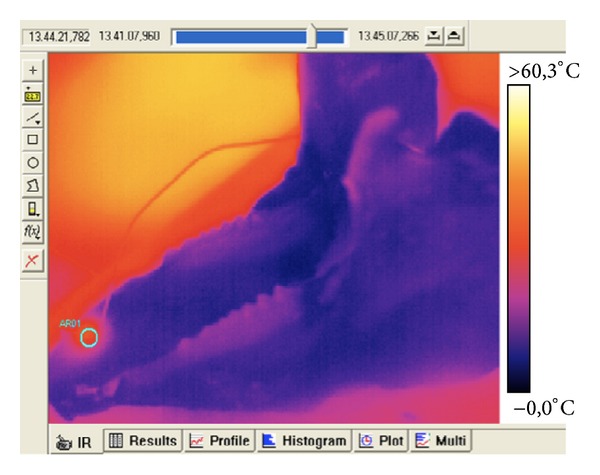
Thermal camera image of the specimen after treatment where the little blue circle defines the laser irradiated area.

The anatomical specimens were placed in front of the thermal camera to detect and to record dynamically the changes in external temperature during the course of surgical interventions (Figure 4).

Temperatures of the anatomical specimen were recorded at the beginning of the test and in correspondence to the highest temperature reached during the intervention (Figure 7).

Before starting with experimental protocol, every laser device was checked with power meter devices (Nova II, Ophir, Jerusalem, Israel) in order to check the real emitted power. For all the used laser devices, the loss of power was between 18% and 25%.

A titanium abutment (Overmed S.R.L., Milano, Italy) was connected to each implant and a 3-grade (ASTM International classification) titanium bar (Panthera Dental, Québec, Canada) of 2 mm diameter was welded to the abutments of each jaw, on one side (for a total of 6 bars), through the use of a resistance spot welder (VISION STRATEGICA Newmed srl, Reggio Emilia, Italy) and, on the other side, by laser device (Fidelis III Plus, Fotona, Ljubljana, Slovenia).

During all the welding procedure, the bone thermal elevation was recorded by the thermal camera and two k-thermocouples.

Due to the necessity to weld titanium under shield atmosphere, argon gas was spread during all the procedures; its utilization, without risks, also “in vivo” in human subjects, has been reported by several authors [22, 23].

**Figure 5:**
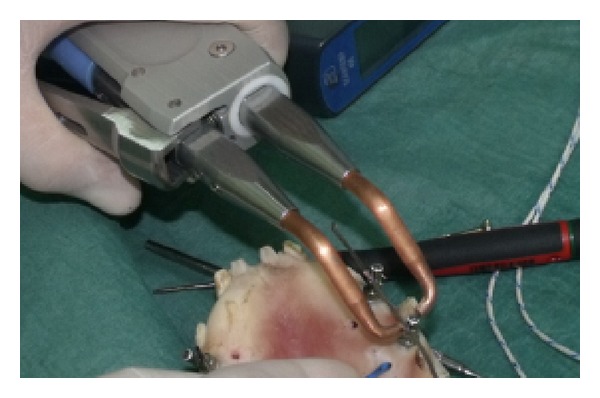
The resistance spot welding process.

The resistance spot welder was used with the following parameters: 25 V, 50 Hz, and 312 J (Figure 5).

The Nd:YAG laser was used with the following parameters: output power 9.85 W, frequency 1 Hz, energy 9.85 J, and pulse duration 15 msec.

**Figure 6:**
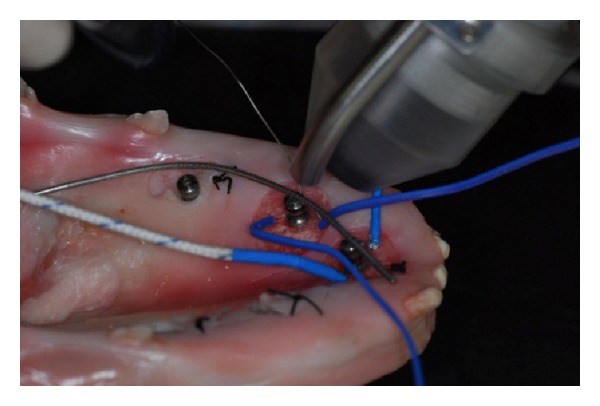
The laser welding process.

**Figure 7:**
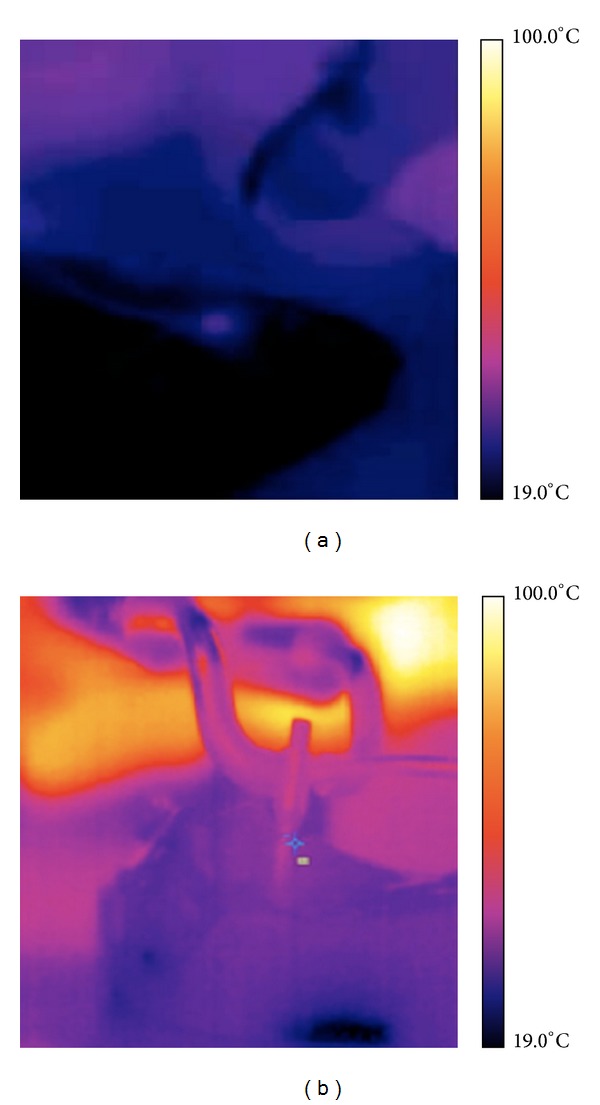
Thermal camera images of laser welding (a) and resistance spot welding (b).

The spot beam obtained with a working distance of 40 mm had a diameter of 0.6 mm, and the fluence was 3300 J/cm^2^ (Figure 6).

Implants were extracted from the jaws by clamping each of them with a narrow plier without touching the bar and the quality of the strength joint was evaluated by traction tests.

**Figure 8:**
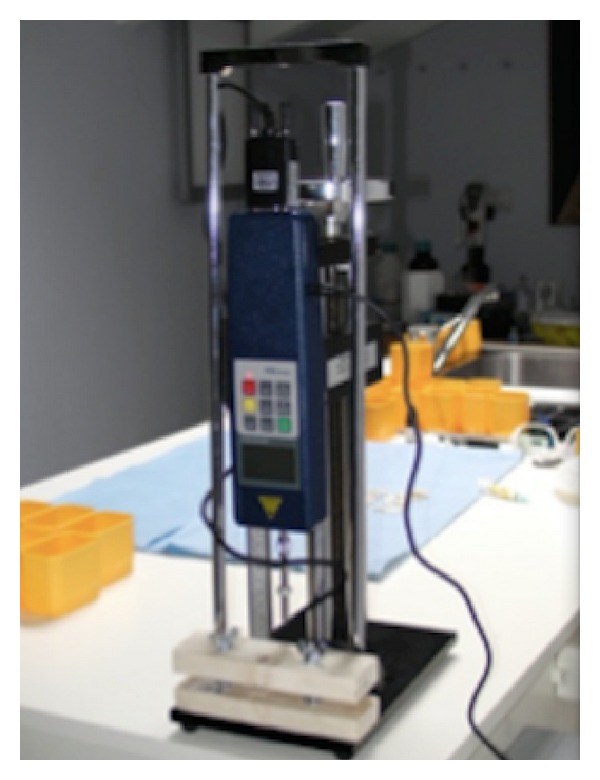
Digital dynamometer used for traction test.

Each implant was clamped to a wise and the point of connection of the bar to the implant was connected to a dynamometer (PCE SH 500-SLJ50 stand PCE Group, Lucca, Italy) mounted on a manual stand in order to record the force (in N) necessary to detach the bar from the abutment (Figure 8).

Subsequently, a statistical analysis using “values unpaired *t*-test” was performed to compare and integrate the result recorded.

## 3. Results and Discussion

**Figure 9:**
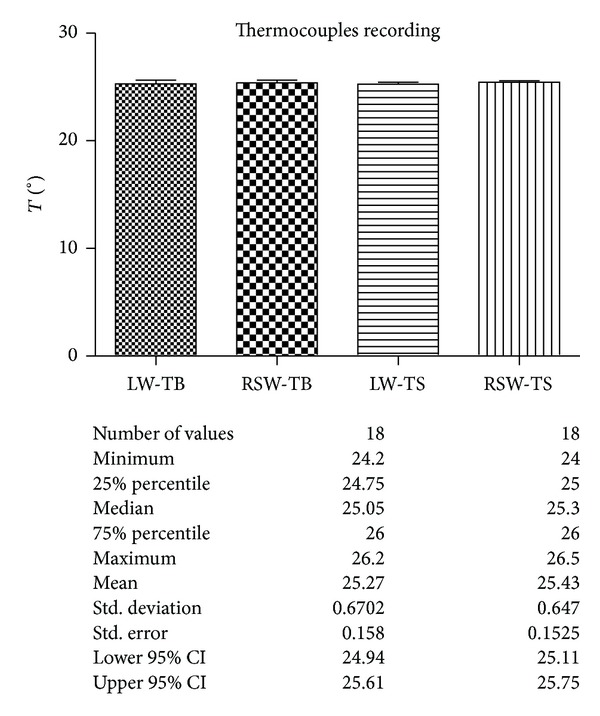
Comparison of the temperature in the bone (TB) and in the implants (TS) recorded by K-thermocouples during laser welding (LW) and spot resistance welding (RSW). Description of the statistical analysis used is shown below.

The analysis of the recorded values by thermocouples is shown in Figure 9.

**Figure 10:**
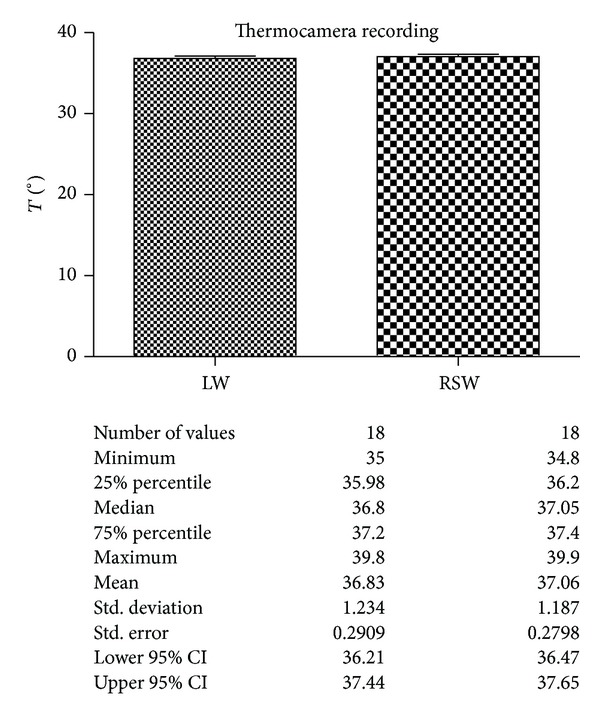
Comparison of the temperature in the surface recorded by thermal camera during laser welding (LW) and spot resistance welding (RSW). Description of the statistical analysis used is shown below.

The analyses of the recorded values by thermal camera are shown in Figure 10.

**Figure 11:**
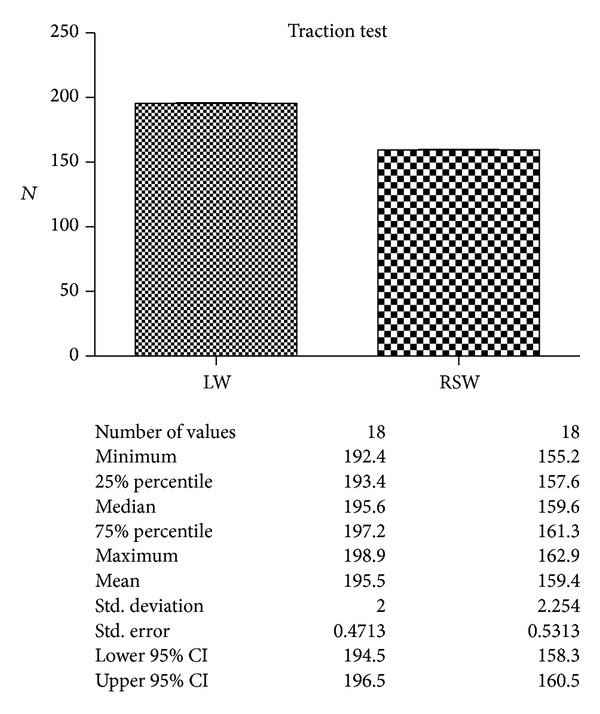
Comparison of the strength in the two groups. Description of the statistical analysis used is shown below.

The analyses of the values obtained by the traction test are shown in Figure 11.

The results obtained by the thermal elevation recorded by k-thermocouples and thermal camera showed a very slight difference between the two groups, not statistically significant.

The strength between the two groups evidenced by traction test showed a great difference, statistically very important, with the best results obtained by the laser welding.

Currently, only two techniques are described as potentially being able to weld metals intraorally, the resistance spot welding and the Nd:YAG laser welding, and a comparative analysis among these two techniques can be difficult mainly on the basis of the great physical differences. Authors in a previous “in vitro” study [24] compared the strength of the joints laser welded and resistance spot welded by a dynamometer on CrCoMo plates, showing a significant difference.

Laser technology is the most efficient method for delivering thermal energy to small areas and, according to many authors [25, 26], it is one of the best fusion welding techniques for different metals. This depends on the possibility to focus the light beam in a focal point. Such a beam delivers energy into the metal causing an increase of temperature which is able to liquefy the material metal-heat. The metal evaporates and a cavity is formed immediately under the heat source and a reservoir of melted metal is produced around it. As the heat source moves forward, the hole is filled with the melted metal from the reservoir and this solidifies to form the weld bead [27]. The best advantage is that the weld process can usually be performed exactly where it is required, that is, at the level of an implant abutment.

Moreover, the procedure can be carried out directly on the master cast, thereby eliminating the risk of inaccuracies and distortions due to the duplication of the model [28] and the heat source, being a concentrated high-power light beam, and minimalizing the distortion problems in the prosthetic pieces [29]. The process allows the possibility of welding in proximity to acrylic resins or ceramic parts with no physical (cracking) or colour damage [30] and these characteristics diminish the working time through the elimination of the necessity for the remaking of broken prosthetic or orthodontic appliances. Several works based on laboratory tests have concluded that laser-welded joints have a high, reproducible strength [31].

In order to stabilize implant abutments “in vivo,” Mondani and Hruska proposed, in the 80s, a welding machine which works through the current impulse at very high voltage, for a very short time, thus allowing the interdigitations of the titanium prisms, resulting in a welding through a process called “syncrystallization” [32, 33].

In physics, the “syncrystallization” is the union of two metal surfaces by sharing the atoms when building the crystal lattice in the junction zone while “electric resistance welding” is an autogenic welding procedure through the employment of pressure, where the heat necessary to reach the melting or forging temperatures is supplied by an electric arch [34].

In the welding cycle, three phases are identified: (1) the joining phase where pressure alone with no current is applied; (2) the welding phase with the simultaneous actions of pressure and current, and (3) the cooling phase where the current is cut off and the pressure only is kept [35].

In the literature, there are no studies about the thermal increase close to the implants during the welding process, whatever is the technique used. Such an aspect should be considered as a key factor, considering the great role of the temperature in the long-term success of the implant-prosthetics therapy.

Unlike industrial solders that can operate only in the presence of argon and without oxygen in the atmosphere, the resistance spot welder used in dentistry can work in the presence of oxygen, water, physiological oral fluids, and blood [36].

The limits of this technique are the capacity to weld only some kind of metals and alloys, the impossibility to be used on patients with pacemakers, and the potential damage to the surrounding structures (teeth, acrylic, and ceramic) [37].

The intraoral laser welding (ILW) technique is effective on all metals and alloys and it can be applied either with or without filler metal and shielding gas; because of the small spot size of the beam (0.6 mm), it is able to restrict the high temperature within a very limited area. In a previous work, the authors demonstrated by an “in vitro” study on plates positioned over the teeth in bovine jaws that, during the laser welding process, the temperature at the surrounding structures level, that is, pulp chamber, is very low and biologically harmless [38] and this work may be considered as a completion and integration of the investigation.

Regarding resistance spot welding, even if many clinical cases are described in several works [35, 39, 40], the paucity of “in vitro” studies about the physical mechanisms and thermal elevations in the biological tissues still makes it very difficult to make a clear point of view on this procedure; this represents an important limit because the possibility to cause an overheating in the tissues represents a real risk in these kinds of situations.

For this reason, the importance of this “ex vivo” consists in the analysis of the biological possible damage by using this technique, with results demonstrating that it is not free of risks.

Literature reports an increase of temperature for dental pulp vitality of 5.5°C during laser treatments in conservative, prosthetic, or aesthetic dentistry as critical [41–43]; moreover, it has been reported that cellular death is immediately valuable with temperature above 70°C [44, 45] and that bone necrosis is caused by a temperature of 47°C for 5 min, 50°C for 1 min, or 56°C for less than 1 min.

This is also considered, in literature, the limit to not exceed in order to achieve the complete bone integration of the implants and to guarantee the complete long-term success of the implant-prosthetic treatment [46–52].

Considering the results obtained, we could consider both techniques used in the tests as free from risks for the safety of the biological structures and being able to be used also for “in vivo” tests.

In fact, the thermal elevation values recorded in the study were greatly below those considered dangerous for the bone health.

Regarding the quality of the welded joint, these tests evidenced the same results of the literature. In fact, a previous “in vitro” study [24] made on CrCoMo plates showed that the quality of the resistance spot welding, from the point of view of the strength, is lower than the laser welding, giving an important indication to the clinic.

## 4. Conclusion

This “ex vivo” study confirmed that both techniques utilized are able to make an effective welding process on the titanium implants.

In particular, the laser welding process seems to give a modest thermal elevation in the bone close to the implants, by far lower than the values, as described above, considered dangerous for their integration and the long-term success of the prosthetic rehabilitation.

Moreover, also the physical characteristics, such as the strength in the joints, presented result which may give a great importance to the technique in the field of implant-prosthetics rehabilitations.

Laser welding may be used also intraorally to connect titanium implant abutments within the technique of the immediate load, without the risk of thermal increase into the bone and with good results in terms of mechanical strength, while the resistance spot welding demonstrated having, beyond the limits above described such the possibility to weld only with an overlapping of the two portions, only few kinds of metal and alloy and only without filler, no advantages in term of thermal elevation and strength.

To complete this study and to verify the results obtained it will be necessary to carry out this investigation by “in vivo” tests and this will be the next step of the topic.
